# Comparison of the Short-Term Effect of Coffee, Energy Drink, and Water on the Eyes in Young Healthy Subjects

**DOI:** 10.7759/cureus.48335

**Published:** 2023-11-05

**Authors:** Güvenç Toprak, Yunus Alkan

**Affiliations:** 1 Ophthalmology, Abant Izzet Baysal University Hospital, Bolu, TUR

**Keywords:** optical coherence tomography, water drink test, energy drink, coffee, caffeine, choroidal thickness

## Abstract

Purpose: We aim to compare the short-term effects of energy drink (ED), coffee, and water on the eyes of young healthy male subjects.

Materials and methods: The right eyes of 30 healthy male subjects were included in this study. We measured the intraocular pressure (IOP), mean arterial pressure (MAP), retinal thickness (RT), choroidal thickness (CT), and retinal nerve fiber layer (RNFL) thickness using spectral domain optical coherence tomography (SD OCT). The measurements for RT and CT were taken at the fovea as well as 1,500 µm nasal and temporal to the fovea. The measurements of the subjects were performed on the first day before water consumption and at 30 minutes and 60 minutes following intake of 250 mL of water. Measurements were repeated at the same regime on the second day after drinking 250 mL of coffee containing an equal concentration of caffeine in ED (37.5 mg) and on the third day after drinking 250 mL of ED. Repeated measures one-way analysis of variance test was used for statistical analysis.

Results: No significant difference was found for ocular perfusion pressure (OPP), MAP, RT, and IOP between the measurements taken on three consecutive days (p>0.05 for all). The CT values for the central, nasal, and temporal segments were significantly reduced in 0-30 and 0-60 minutes following coffee and ED intake (the range of p-value was <0.001-0.027).

Conclusions: Both coffee and ED intake caused acute and significant decreases in CT that persisted for one hour in young healthy male subjects. The impact of ED intake on CT was attributed mainly to its caffeine content.

## Introduction

Energy drinks (EDs) constitute one of the fastest-growing segments of the beverage market, and they are promoted as tasty, alcohol-free, stimulating drinks. EDs contain variable amounts of caffeine, sugar, taurine, glucuronolactone, vitamins, minerals, and various stimulating herbal derivatives, such as guarana, ginseng, and ginkgo biloba, and the exact amounts of the ingredients are generally not disclosed by the producers [1]. Recently, the popularity of EDs has increased significantly, especially among adolescents and young adults, because of intense advertisements stating increased physical and cognitive performance. However, a growing body of evidence suggests the adverse effects of EDs, especially on the cardiovascular system [2].

The systemic adverse effects of EDs are examined in various studies, and their effects are attributed mainly to high caffeine content [2-5]. Although healthy people can tolerate caffeine up to a certain degree, a higher amount of caffeine has been shown to be associated with stroke and seizures [6]. Guarana extract in ED, which contains caffeine, theobromine, and theophylline, can potentiate the effect of caffeine further [5]. Likewise, glucuronolactone also has adverse cardiovascular effects, including elevated platelet aggregation, endothelial dysfunction, and a rise in blood pressure (BP) [7]. The health impacts of the many ED ingredients are still understudied, and unfortunately, the production of ED is not regulated. As a result, this mandates research on the effect of ED on the eye.

The effect of caffeine alone on intraocular pressure (IOP) and choroidal thickness (CT) was examined in some studies [8-11]. Previous reports demonstrated that maximum IOP has been reached 15 and 30 minutes following an intake of a beverage containing caffeine, and the effects persisted for at least 90 minutes afterward [8,12]. It was also reported that caffeine ingestion did not increase IOP significantly in healthy subjects [12]. In addition, several studies have shown that caffeine intake can induce transient CT thinning in healthy individuals. A decline in CT starts five minutes after ingestion of caffeine and continues for about four hours [9-11]. The possible effects of EDs on CT and choroidal blood flow are also investigated in some recent studies [13-15]. Doğan et al. [13] and Mete et al. [14] have speculated that the subfoveal CT does not show any significant thinning between consecutive measurements, whereas Arej et al. [15] have stated that CT changes after ED ingestion with a more pronounced reduction in thicker choroids. Despite the presence of contrary results, these studies have concurrence that ingredients other than caffeine in EDs, such as taurine and inositol, can reduce the vasoconstrictor effect of caffeine on choroid tissue. Thus, we aimed to reveal the effects of pure caffeine and ED, including caffeine with some vasodilator substances, on the choroid tissue of the same individuals.

In the current study, we investigated the short-term impacts of ED on the eye and compared its effect with coffee in young healthy male subjects.

## Materials and methods

This prospective study examined the right eyes of 30 healthy male subjects who were recruited from our faculty population at the Faculty of Medicine of Abant İzzet Baysal University. This study was performed in adherence with the tenets of the Declaration of Helsinki and was approved by the local ethics committee of Abant İzzet Baysal University (approval number: 2016/57). Informed consent was also obtained from all participants who volunteered.

The following exclusion criteria were applied: the presence of ocular disease preventing the examination of the corneal and retinal states, having >3 diopters of spherical or cylindrical refractive error, a history of previous ocular surgery, any ocular or systemic disease, smoking, and drinking alcohol. The following inclusion criteria were applied: aged between 20 and 30 years, having the best corrected visual acuity of 20/20 or better, having no chronic ocular disease (e.g., glaucoma and uveitis), and demonstrating normal blood pressure (brachial systolic/diastolic blood pressure of lower than 140/90 mmHg). All measurements of the included subjects were performed between 10:00 am and 12:00 am. It has been known that caffeine remains active in the human body for 3-6 hours [16]. Therefore, participants were asked not to consume any caffeine-containing products including tea, chocolate, or soft drinks for at least 12 hours before the study and during the study period. Subjects were asked to refrain from drinking caffeinated beverages after midnight to minimize the effects of these vasoactive agents on the results. Serum tests were not performed to confirm abstinence from these agents. Subjects were asked not to consume anything for at least one hour prior to the examination and during the examination period. The measurements of the subjects were performed on the first day before water consumption and 30 minutes and 60 minutes following intake of 250 mL of water. On the second day, measurements were followed before consumption of sugar-free coffee and 30 minutes and 60 minutes following intake of 250 mL of sugar-free coffee (Nescafe® Classic) (a portion that contains 2 g of coffee includes 75 mg of caffeine) that contained approximately 1 g of coffee (37.5 mg caffeine). Likewise, on the third day, measurements were performed before consumption of ED and 30 minutes and 60 minutes following an intake of a can of ED (250 mL of Red Bull® ED containing 150 mg/L caffeine, 800 mg/L taurine, 110 g/L sugar, and various vitamins). Subjects were asked to finish their beverages within three minutes. Optical coherence tomography (OCT) measurements were performed using a follow-up scanning protocol of spectral domain (SD) OCT, in which the scanned images taken on the first day before drinking water were used as the reference image.

A detailed ophthalmologic examination that included the best corrected visual acuity, IOP, spherical equivalent (SE), central corneal thickness (CCT), anterior chamber depth (ACD), axial length (AL), retinal thickness (RT), retinal nerve fiber layer (RNFL) thickness, and CT was performed. IOP was measured using Tonopen Avia tonometry (Reichert Technologies, Depew, NY). CCT and AL were measured using Lenstar LS 900 (Haag-Streit AG, Koeniz, Switzerland). Global RNFL (RNFLg) thickness, RT, and CT were measured using the Spectralis SD OCT (Heidelberg Engineering, Heidelberg, Germany).

**Figure 1:**
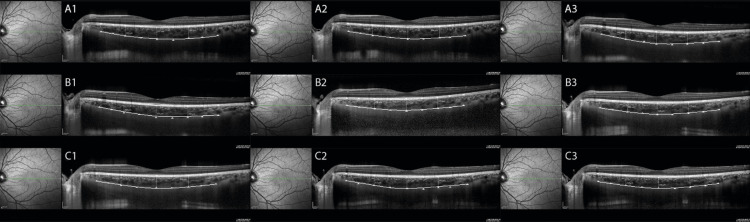
Choroidal thicknesses Choroidal thicknesses at baseline, 30 minutes, and 60 minutes following intake of water (A1, A2, and A3, respectively), coffee (B1, B2, and B3, respectively), and energy drink (C1, C2, and C3, respectively)

All OCT scans and measurements were performed by the same experienced operator (GT) with Spectralis OCT (software version 5.3), which uses the horizontal 30° line scan enhanced depth imaging (EDI) mode through the fovea. The line scan images were saved for analysis after 100 frames were averaged using the automatic real-time (ART) imaging value 100 and active eye-tracking feature. The automatic segmentation values of the Spectralis OCT were used to measure the RT. The CT was measured manually from the outer portion of the hyper-reflective line that corresponds to the retina pigment epithelium to the inner surface of the sclera (Figure 1). CT was measured by two experienced operators (GT and YA) using manual calipers that were provided with the OCT software. The readers were blinded to the experimental groups and were not aware of previous CT measurements or the scanning phase of the subjects. The EDI images of the subjects were examined by another operator (FU), and CT measurements were performed by blinded readers (GT and YA) on different days. For intraobserver reproducibility, GT performed two CT measurements, and for interobserver reproducibility, YA performed one CT measurement. Default RNFL optic disc protocol was used in the study, consisting of a 3.6 mm diameter circle scan for RNFL analysis, using an ART imaging value of 100 and the active eye-tracking feature. RNFLg thickness measurements were performed using the automatic segmentation values of the Spectralis OCT system. OCT scans were acquired without pupil dilation, and image quality below 30 was excluded from the study. The measurements were comprised of the central foveal, 1,500 µm nasal, and 1,500 µm temporal segments from the center of the fovea for CTes at 100% magnification. Fine corrections were performed at 200% magnification.

Automated blood pressure measurement was performed for the examination of the systolic and diastolic blood pressures of the participants (Omron M2 Basic, Omron, Japan). A formula of diastolic pressure plus one-third of the pulse pressure was used to calculate the mean arterial pressure (MAP). Ocular perfusion pressure (OPP) was calculated according to the formula of two-thirds of MAP minus IOP.

Data were analyzed using the Statistical Package for the Social Sciences (SPSS) statistical software version 25.0 (SPSS Inc., IBM Corporation, Chicago, IL). Data were presented as the mean values from each data set ± standard deviation (SD). The first measurements performed by GT were used for the statistical analysis. The normality of the sample distribution was determined using the Shapiro-Wilk test. For all of the measured values, the p-value for the Shapiro-Wilk test was found higher than 0.05 (the actual values ranged from 0.082 to 0.947). The repeated measures analysis of variance test and post hoc Bonferroni test were used for statistical analyses. The Greenhouse-Geisser or Huynh-Feldt correction was applied to the degrees of freedom if sphericity was violated and the Ɛ value for the correction was below or over 0.750, respectively. The coefficient of repeatability (CR) defined by Bland and Altman was also performed for the CT measurements that were performed by two blinded operators in order to determine intraobserver and interobserver reproducibility [17]. p<0.05 was considered statistically significant.

## Results

None of the subjects was a regular consumer of ED. The studied parameters and the statistical significance are shown in Table 1. The mean age and body mass index of the study group were 26.10±1.61 years (range: 24-30 years) and 23.18±2.35 kg/m^2^ (range: 18.17-25.38 kg/m^2^), respectively. The best corrected Snellen visual acuity of the subjects was 20/20. The mean SE, ACD, AL, and CCT were -1.14±1.08 D, 3.28±0.22 mm, 23.85±0.99 mm, and 547.18±25.42 µm, respectively. All subjects have a 1:1 or higher ratio of aqueous gap/corneal thickness according to the Van Herick grading method.

**Table 1:** Measurements of systemic and ocular parameters before and after drinking water, coffee, and energy drink *One-way repeated measures analysis of variance (p<0.05 indicates a statistical significance) CT: choroidal thickness, ED: energy drink, IOP: intraocular pressure, MAP: mean arterial pressure, OPP: ocular perfusion pressure, RNFLg: global retinal nerve fiber layer thickness, RT: retinal thickness, SD: standard deviation

Parameter	Drink	Baseline (mean±SD) (range)	30 minutes (mean±SD) (range)	60 minutes (mean±SD) (range)	p*
IOP (mmHg)	Water	15.747±3.168 (9.8-21.0)	15.987±2.808 (9.5-21.5)	15.350±2.911 (10.1-19.8)	0.324
	Coffee	15.697±2.665 (10.4-21.1)	15.130±2.818 (10.2-22.1)	15.257±2.361 (11.2-19.4)	0.414
	ED	15.643±2.766 (10.2-20.6)	14.973±2.953 (8.1-21.6)	14.940±2.545 (8.4-20.2)	0.170
MAP (mmHg)	Water	90.844±6.013 (78.33-101.33)	89.800±7.665 (69.33-102.67)	89.489±5.379 (80.0-103.67)	0.536
	Coffee	89.889±6.594 (77.33-104.0)	90.778±6.552 (80.0-106.67)	92.000±7.293 (73.33-104.0)	0.242
	ED	89.489±5.263 (75.33-99.0)	89.867±6.597 (76.0-100.0)	87.989±5.612 (72.67-98.67)	0.245
OPP (mmHg)	Water	44.816±5.973 (35.42-55.96)	43.880±5.905 (28.02-55.03)	44.280±5.073 (34.73-55.68)	0.585
	Coffee	44.229±4.923 (33.81-53.14)	45.389±4.210 (39.28-54.78)	46.077±5.084 (35.29-54.91)	0.122
	ED	44.016±3.519 (37.07-51.10)	44.938±5.547 (32.97-56.34)	43.719±4.246 (34.36-50.49)	0.266
Foveal RT (µm)	Water	219,130±10.789 (201-247)	219.230±11.294 (202-246)	218.870±11.702 (202-247)	0.696
	Coffee	219.100±10.921 (202-247)	219.100±11.000 (202-246)	219.033±11.385 (202-245)	0.931
	ED	219.900±12.557 (200-252)	220.100±11.309 (202-248)	218.670±12.138 (201-251)	0.089
Temporal RT (µm)	Water	335.170±17.112 (303-366)	335.200±17.004 (299-362)	335.370±16.091 (302-363)	0.950
	Coffee	336.100±16.800 (302-362)	336.033±17.109 (298-364)	336.167±16.783 (301-363)	0.892
	ED	335.800±17.105 (303-365)	336.230±17.614 (300-364)	335.970±16.428 (298-363)	0.721
Nasal RT (µm)	Water	350.000±18.105 (312-381)	348.230±16.815 (312-375)	348.100±17.652 (311-378)	0.095
	Coffee	349.300±16.906 (313-376)	348.767±16.548 (314-375)	349.000±16.457 (316-374)	0.288
	ED	349.000±16.950 (313-381)	348.700±17.923 (312-380)	348.700±17.525 (312-381)	0.905
RNFLg (µm)	Water	98.570±7.829 (81-115)	98.470±8.140 (81-115)	98.170± 7.720 (81-115)	0.577
	Coffee	98.667± 7.331 (83-114)	98.600± 7.668 (82-116)	98.133± 7.546 (81-114)	0.035
	ED	98.33±7.980 (83-115)	98.230± 7.641 (81-113)	97.930±7.506 (81-114)	0.312
Subfoveal CT (µm)	Water	343.900±58.371 (230-552)	341.43±62.509 (226-558)	337.630±64.273 (210-551)	0.005
	Coffee	343.700±61.372 (210-550)	338.233±60.732 (205-538)	329.800±62.695 (190-532)	<0.001
	ED	343.200±61.357 (216-556)	334.400±59.657 (210-534)	327.70±60.536 (215-541)	<0.001
Temporal CT (µm)	Water	318.600±70.515 (201-511)	317.40±70.187 (225-507)	316.300±68.658 (217-512)	0.465
	Coffee	315.767±70.599 (215-530)	313.967±69.919 (217-525)	311.667±70.840 (207-523)	0.017
	ED	314.600±70.539 (217-519)	308.13±73.103 (216-511)	306.400±70.134 (209-514)	0.001
Nasal CT (µm)	Water	316.530±65.679 (199-502)	315.87±62.715 (201-488)	314.67±62.891 (203-490)	0.595
	Coffee	313.333±60.640 (196-465)	311.733±60.600 (206-475)	308.533±62.837 (198-482)	0.027
	ED	315.87±64.837 (198-488)	308.600±63.733 (195-457)	307.330±66.765 (192-478)	0.002

The repeated measures analysis of variance test using Bonferroni adjustment demonstrated a significant change for all CT segments following coffee or ED intake (the range of p-value was <0.001-0.027) (Table 1). The CT continued to decrease after water, coffee, or ED intake, but this decrease was more prominent following the intake of coffee or ED. RT, IOP, MAP, and OPP measurements did not reveal any significant change (the range of p-value was 0.089-0.950) (Table 1). There was a statistically significant but clinically insignificant difference in RNFLg thickness after drinking coffee (p=0.035) (Table 1). However, RNFLg values were similar following water or ED intake (p-values were 0.557 and 0.312, respectively) (Table 1).

A pairwise comparison with Bonferroni adjustment revealed statistically significant differences in the subfoveal CT measurements after drinking water between 0 and 60 minutes (p=0.021) and 30 and 60 minutes (p=0.040), after drinking coffee between 0 and 30 minutes (p=0.004), 0 and 60 minutes (p<0.001), and 30 and 60 minutes (p<0.001), as well as after drinking ED between 0 and 30 minutes (p=0.002), 0 and 60 minutes (p<0.001), and 30 and 60 minutes (p=0.003) (Figure 1). A pairwise comparison with Bonferroni adjustment showed a significant change for temporal CT measurements after drinking ED between 0 and 30 minutes (p=0.040) and 0 and 60 minutes (p=0.002). However, temporal CT measurements were statistically not significant after drinking coffee at the time points tested (the range of p-value was 0.052-0.331) (Figure 1). A pairwise comparison with Bonferroni adjustment showed a significant change for the nasal CT measurements between 30 and 60 minutes following coffee intake (p=0.033) and between 0 and 30 minutes (p=0.039) and 0 and 60 minutes (p=0.005) following ED intake (Figure 1).

Evaluation of intraobserver and interobserver differences in subfoveal CT measurements using the Bland and Altman method revealed that there was no statistically significant difference between the measurements of the operators (GT and YA). The coefficient of repeatability (CR) for subfoveal CT, nasal CT, and temporal CT measurements ranged between 13.85 and 19.42 µm for intraobserver measurements (the range of p-values was 0.103-0.416) and 17.81-24.92 µm for interobserver measurements (the range of p-values was 0.086-0.347).

## Discussion

To our knowledge, this is the first study that compared the impact of coffee and ED on the eye, and we found that ED caused a significant CT decrease within 60 minutes following ED consumption. The effect of ED on CT was similar to caffeine. ED did not significantly alter the IOP, RT, and RNFL thickness. Coffee did not alter IOP and RT, but it caused a significant change in RNFL thickness when measured at 60 minutes following its consumption. However, this change in RNFL thickness was clinically insignificant. Moreover, ED and coffee did not cause any significant change in MAP and OPP. Ingestion of a can of ED did not have any significant effect on IOP, OPP, or MAP among healthy male participants.

Caffeine mainly affects the cardiovascular system, where it causes myocardial stimulation, vasoconstriction, and an increase in BP. It also significant impacts on the central nervous system in humans [18]. ED containing 80 mg of caffeine elevates both diastolic and systolic blood pressure [19]. This is higher than the effects of caffeine intake alone, which suggests a possible interaction between caffeine and other components of EDs [19]. Blood pressure is reduced in both hypertensive animals and humans following taurine treatment [20,21]. Likewise, its deficiency leads to increased blood pressure [21,22]. While caffeine elevates blood pressure, both animal and clinical studies suggested that blood pressure is decreased after taurine treatment [23]. In another study, increased activity of the sympathetic nervous system due to elevated plasma adrenaline and noradrenaline levels following ED consumption was primarily attributed to the caffeine content of ED [24]. Conversely, it was also reported that 250 mL of ED did not cause any significant change in systolic or diastolic blood pressure [25-27]. In the present study, healthy young subjects, who consumed only 250 mL of ED containing 37.5 mg of caffeine, did not show any significant acute effect on MAP and eye other than CT. Similar to the results of the current study, sugar-free ED has nearly the same effect as caffeine-equivalent water controls, which indicates that other contents of ED have limited or no effect [28].

At a dose of 200 mg, caffeine reduced retinal blood flow by 13%, and higher doses caused vasoconstriction [29]. In another study, 100 mg of caffeine decreased chorioretinal blood flow by 6% at 60 minutes [30]. In retrobulbar hemodynamic studies, oral intake of caffeine by healthy volunteers caused a significant increase in the resistive index of the ophthalmic, central, and short posterior ciliary artery that was detected by color Doppler ultrasonography [31]. It was reported that drinking a cup of coffee containing 57 mg of caffeine caused a significant decrease in CT for at least four hours [10]. Similarly, Zengin et al. [11] found a reduction in CT after 200 mg caffeine intake. The changes in CT following the consumption of coffee were directly correlated with age [10]. This fact might be explained by the reduced metabolic rate with advanced age and the more pronounced effects of caffeine in the elderly [10]. In the current study, a lower amount of coffee was used, but the impact on CT was still significant. In accordance with previous studies, choroidal thinning following the consumption of coffee might be associated with an increase in the vascular resistance of the blood vessels and a decrease in ocular blood flow.

Besides the studies suggesting that caffeine thins the choroid and alters ocular blood flow, different consequences have been observed in studies regarding the effect of ED on the choroid [10,11,13-15,29-31]. It has been found that RT, CT, and RNFL thickness are not affected by ED intake, whereas CT increased 30 and 60 minutes after consumption of 250 mL of water [13]. Moreover, in OCT angiography measurements, an increase in vascular density of the parafoveal and perifoveal deep capillary plexus was observed after ED intake [13]. Similarly, Mete et al. [14] stated that ED has no significant thinning effect on subfoveal CT. In conclusion, it has been speculated that other agents in EDs may reduce the vasoconstrictive effect of caffeine on choroidal vessels. Furthermore, agents such as taurine and inositol are claimed to have a vasodilatory effect that surpasses the vasoconstrictive effect of caffeine [13,14]. However, Arej et al. [15] have reported that ED consumption reduces subfoveal CT significantly, despite the possible vasodilatory effect of taurine. They have shown a 14 µm thinning in subfoveal CT, similar to our results [15]. We also found a significant decrease in subfoveal, nasal, and temporal CTes in both caffeine and ED consumption. These different results might be caused by the measurement methods of CT. The authors have not detailed the CT measurement methods and statistics evaluating intraobserver and interobserver reproducibility of CT measurements in their studies [13,14]. Furthermore, the effects of caffeine and ED on CT were similar in the present study. Therefore, the main effect of ED on the eye can be attributed to caffeine. However, the isolated evaluation of agents other than caffeine in ED is still necessary to enlighten the exact effect of ED ingredients on the eye.

The effect of caffeine on IOP is controversial [32-34]. IOP levels in patients with ocular hypertension, open-angle glaucoma, and healthy volunteers were investigated after caffeine intake, and it was found that those with glaucoma had significantly greater elevation in IOP (approximately 3 mmHg) [32,35]. Moreover, before elevations in IOP, blood pressure is increased in response to acute caffeine intake [36,37]. After caffeine intake, blood flow is decreased to both the macula [30] and the optic nerve head, as well as to the choroid and retina [29], which results in elevated susceptibility of the optic nerve to increased IOP [38]. In another study, it was reported that 182 mg of caffeine statistically increased IOP, but it did not clinically impact IOP in patients with or at risk for primary open-angle glaucoma [39]. Li et al. [12] reported that IOP was not changed by ingestion of caffeine in healthy subjects; however, IOP was increased significantly in patients with glaucoma or ocular hypertension. These results are in parallel with our findings, which suggest that ED or coffee containing 37.5 mg of caffeine has no significant effect on IOP.

Nowadays, the caffeine content in EDs is similar to many other coffee beverages. However, ED consumption is generally higher than the consumption of one can, which was the amount used in the current study. In a study that included 496 college students, it was reported that 51% of them regularly consumed more than one ED/month, and the majority of them habitually drank EDs several times per week [40]. Higher doses of caffeine, repeated intake, and habitual caffeine consumption generally result in increased half-life and reduced clearance of caffeine and its metabolites and, as a result, lead to an elevated potential for toxicity [41]. We included healthy young subjects who were not regular consumers of ED. Thus, the actual effect of ED on the eye might be stronger than observed in the current study.

The current study has some limitations. We included young, healthy subjects, but not those at risk of cardiovascular diseases. We also used only 250 mL of ED, which contains only a small amount of caffeine, and this might have a significant impact on the results. Companies are constantly changing the concentrations of ED ingredients, which in turn causes a significant variation of ingested ingredients, especially caffeine. There are also many factors that could impact the results and cause discrepancies between different studies, including variations in lifestyle and feeding habits of the subjects, state of fitness (trained versus untrained), and variations in type, amount, and active metabolites in different EDs, as well as variations in the consumed amount of even same type and ingredients of ED. Our CT measurements were performed manually because there is no software algorithm that exists for choroidal segmentation. However, our study had certain advantages. CT measurement was done at three different segments to better evaluate CT changes. We performed all OCT scans at the same time of the day to avoid diurnal fluctuations in CT [42]. Another advantage is the exclusion of the adverse effects of any medication, cigarette, or alcohol from the results since none of the volunteered subjects used any of these substances. Disposing of the effects of systemic or topical medications and diurnal variation on CT makes the results of this study more valuable.

## Conclusions

In conclusion, to our knowledge, this is the first study comparing the effects of ED and coffee on the eye. The results demonstrated that ED and coffee caused a decrease in CT at 30 minutes of drinking that gradually continued to decrease after 60 minutes in healthy young males. Although it was hard to link the effects of ED to a specific ingredient due to the variations in ingredients, in the current study, the effects of ED on the eye are thought to be mainly associated with the caffeine content of ED. The effect of ED on CT and other ocular parameters might differ according to age and higher amount of ED consumption. Therefore, the dose-dependent influence of ED on IOP, CT, and blood pressure needs further investigation. Further prospective studies with larger sample sizes, different ages, and various patient groups are required for a better conclusion for the potential effects of ED on the eye by considering the substantially increasing popularity of ED.
